# Altered Small-World Networks in First-Episode Schizophrenia Patients during Cool Executive Function Task

**DOI:** 10.1155/2018/2191208

**Published:** 2018-09-05

**Authors:** Zongya Zhao, Yaqing Cheng, Zhenxin Li, Yi Yu

**Affiliations:** ^1^School of Biomedical Engineering, Xinxiang Medical University, Xinxiang 453003, China; ^2^Key Lab of Neurosense and Control, Xinxiang Medical University, Xinxiang 453003, China; ^3^Department of Computer Technology, Henan Information Engineering School, Zhengzhou 450000, China

## Abstract

At present, little is known about brain functional connectivity and its small-world topologic properties in first-episode schizophrenia (SZ) patients during cool executive function task. In this paper, the Trail Making Test-B (TMT-B) task was used to evaluate the cool executive function of first-episode SZ patients and electroencephalography (EEG) data were recorded from 14 first-episode SZ patients and 14 healthy controls during this cool executive function task. Brain functional connectivity between all pairs of EEG channels was constructed based on mutual information (MI) analysis. The constructed brain functional networks were filtered by three thresholding schemes: absolute threshold, mean degree, and a novel data-driven scheme based on orthogonal minimal spanning trees (OMST), and graph theory was then used to study the topographical characteristics of the filtered brain graphs. Results indicated that the graph theoretical measures of the theta band showed obvious difference between SZ patients and healthy controls. In the theta band, the characteristic path length was significantly longer and the cluster coefficient was significantly smaller in the SZ patients for a wide range of absolute threshold *T*. However, the cluster coefficient showed no significant changes, and the characteristic path length was still significantly longer in SZ patients when calculated as a function of mean degree *K*. Interestingly, we also found that only the characteristic path length was significantly longer in SZ patients compared with healthy controls after using the OMST scheme. Pearson correlation analysis showed that the characteristic path length was positively correlated with executive time of TMT-B for the combined SZ patients and healthy controls (*r* = 0.507, *P* = 0.006), but not for SZ patients alone (*r* = 0.072, *P* = 0.612). The above results suggested a less optimal organization of the brain network and could be useful for understanding the pathophysiologic mechanisms underlying cool executive dysfunction in first-episode SZ patients.

## 1. Introduction

Schizophrenia (SZ), one of the most serious mental disorders, usually causes many aspects of cognitive dysfunction, including memory, attention, and executive function [1]. And executive dysfunction is considered to be one of the most critical cognitive dysfunctions [2]. Generally, executive function is involved in a range of higher-level cognitive processes including anticipation, goal selection, planning, behavior's choice, inhibition, self-control, and self-monitoring [3, 4]. At present, a large number of studies have shown that patients with schizophrenia are accompanied by severe executive dysfunction [5–8].

It has been demonstrated that the cognitive dysfunction or other symptoms of SZ can be interpreted in terms of altered brain functional connectivity among different brain regions [9]. A large number of electroencephalography (EEG) and functional magnetic resonance imaging (fMRI) studies have confirmed dysfunctional connectivity in SZ patients [10–16]. Among the usually applied methods to study functional connectivity, such as coherence and correlation coefficient, mutual information (MI) has been widely applied in many studies to investigate the information communication and connectivity among different brain regions [11, 17, 18]. MI is based on information theory [19] and can be used to measure the amount of information that can be obtained about one variable from the measurement of another. Different from the traditional correlation coefficient which only measures linear dependence between time series, MI takes account of both linear and nonlinear dependencies, which makes MI a superior method for studying dynamical coupling or information transmission between EEG data. Some researchers tried to use MI to study schizophrenia, but the results do not seem consistent [11, 17, 20].

In recent years, graph theory analysis has been widely applied to study the topologic characteristics of brain functional networks. Previous studies have implied that the small-world network is considered to be one of the most appropriate models to balance local segregation and integration in the human brain [21, 22]. The small-world network is characterized by a higher cluster coefficient compared to a random network and a shorter path length compared to a regular network, which allows for more efficient information transfer among distant brain regions. Previous EEG studies have indicated that SZ patients showed a disrupted small-world network in the rest state [23, 24], during working memory task [25–27], during oddball paradigm task [28, 29], and in processing local contextual information [30]. For example, Shim et al. [28] found that reduced cluster coefficients and increased path lengths appeared in SZ patients during an oddball task. Therefore, we hypothesized that the disrupted small-world network would also appear in SZ patients during a cool executive function task.

Because executive function impairment is considered to be one of the most critical cognitive dysfunctions, it is of importance to investigate the brain functional networks in SZ patients during an executive function task. Zelazo and Müller [31] divided executive function into cool executive function and hot executive function. The hot executive function is related to emotional involvement and needs flexible evaluation about the emotional significance of the stimulus, whereas the cool executive function is unrelated to emotional involvement and often caused by decontextualized tasks, because the cool executive function is not involved in emotional arousal and clinical observations have shown that emotional reactions of SZ patients usually do not match their inner experience [32]. At present, some cool executive function tasks including Trail Making Test-A (TMT-A) and Trail Making Test-B (TMT-B) have been applied to evaluate the cool executive function of schizophrenia patients [33, 34]. However, little is known about the brain functional connectivity and its small-world topologic properties in first-episode schizophrenia patients during a cool executive function task.

By taking all these considerations into account, the aim of the present study was to combine functional connectivity based on MI with graph theory analysis to investigate the brain functional network in first-episode SZ patients during a cool executive function task. In this paper, the functional connectivity matrixes were constructed by using MI analysis between all pairs of EEG channels in different frequency bands. Then, the constructed brain functional networks were filtered by three thresholding schemes: absolute threshold, mean degree, and a novel data-driven scheme based on orthogonal minimal spanning trees (OMST), and graph theoretical measures were calculated. The differences between SZ patients and healthy controls were evaluated by statistical analysis. Finally, Pearson's correlation was used to evaluate the relationship between cluster coefficient *C* or path length *L* and task performance.

## 2. Materials and Methods

### 2.1. Subjects

14 first-episode SZ patients (9 male and 5 female) were recruited from the Henan Psychiatric Hospital of China according to the Structured Clinical Interview for DSM-IV, and any patients with a history of medication treatment, drug abuse/dependence, electroconvulsive therapy, or other psychiatric and neurological diseases were excluded. The mean age of the 14 SZ patients was 28.21 ± 6.94 years, the mean duration of illness was 18.26 ± 7.03 months, and all patients are right-handed. For healthy controls, a group of 14 subjects matched for sex, age, and dominant side was recruited (9 male and 5 female; mean age: 25.13 ± 3.75; all right-handed), and any healthy controls with a past or current psychiatric illness, drug dependence, neurological disorders, or severe somatic diseases were excluded. The study was approved by the ethics committee of Henan Psychiatric Hospital of China, and an informed consent form was signed by all participants before the experiment.

### 2.2. EEG Recordings and MI Computation

The EEG data were recorded from all participants when they were performing a cool executive function task, that is, the TMT-B task. As for the TMT-B task that evaluates quick visual search, visual space sorting, and cognitive set transfer functions, the participants were asked to connect numbers (1–13) and letters (A–M) using a pen on a paper according to an alternating sequence as fast as possible, and the pen tip cannot leave the paper during this process. The execution time to complete this task and the error number that is the number of incorrectly linked numbers and letters were used to evaluate the task performance of the participants.

The EEG data were recorded at a sampling rate of 1000 Hz from 24 channels (FP1, FPz, FP2, AF3, AF4, F7, F5, F3, F1, Fz, F2, F4, F6, F8, T7, C3, C4, T8, P7, P3, P4, P8, O1, and O2) that were mounted on the scalp with a 64-channel EEG cap according to the 10-20 standard system. And the impedance of all electrodes was below 10 kΩ.

Offline EEG preprocessing was carried out by using Matlab 7.7.0 R2010a software (Mathworks Inc., USA) equipped with the EEGLAB toolbox [35]. Firstly, a 0.5–30 Hz zero-phase bandpass filter was applied. Then, ocular and prominent muscle artifacts were removed by means of independent component analysis (ICA), and the average number of artifactual independent components was 3.1 ± 0.8 (mean ± std) and 3.4 ± 0.7 (mean ± std) for healthy controls and SZ patients, respectively. Subsequently, the EEG data were divided into 10-second epochs and recomputed against the average reference. Finally, the following 4 frequency bands were obtained using a zero-phase bandpass filter: delta (0.5–3 Hz), theta (4–7 Hz), alpha (8–13 Hz), and beta (13–30 Hz).

MI is based on information theory and can be used to measure the amount of information that can be obtained about one variable from the measurement of another. The main advantage of MI is that it takes account of both linear and nonlinear dependencies. The detailed calculation method of MI was described in some previously published literature [11, 17, 18, 36]. Briefly, given two random variables *X* and *Y*, the pairwise MI is defined as

(1) $$\begin{array}{c} MI\left(X,Y\right)=\underset{x,y}{\sum }P_{XY}\left(x,y\right)\log_{2}\frac{P_{XY}\left(x,y\right)}{P_{X}\left(x\right)P_{Y}\left(y\right)}, \end{array}$$

where *P*_*X*_(*x*) is the probability that *x* is drawn from *X* and  *P*_*XY*_(*x*, *y*) is the joint probability density function for the measurements of *X* and *Y* that produce the values *x* and *y*.

MI is estimated from a finite number of samples, and the probability densities,  *P*_*X*_(*x*) and *P*_*XY*_(*x*, *y*), are approximated by histogram (using bin size of 100). For a fair comparison across subjects and frequency bands, here we computed the normalized MI as

(2) $$\begin{array}{c} Normalized\;MI\left(X,Y\right)=\frac{MI\left(X,Y\right)}{H\left(X\right)+H\left(Y\right)}, \end{array}$$

where *H*(*X*) and *H*(*X*) are the entropies and *H*(*X*) is defined as ∑_*x*_*P*_*X*_(*x*)log_2_*P*_*X*_(*x*). The normalized MI is in the range of [0, 1]. Here, the data are separated into 10-second epochs for MI computation in order to increase the sample size as well as to enhance the stationarity and consistency of the MI computation.

MI between all pairs of EEG channels was computed, resulting in a 24 × 24 matrix (24 is the number of EEG channels). For each epoch, the MI matrix was computed, and an average MI matrix for each subject was obtained by averaging the MI matrixes calculated from all epochs. According to the above process, the MI matrixes for the above-mentioned four frequency bands (delta: 0.5–3 Hz, theta: 4–7 Hz, alpha: 8–13 Hz, and beta: 13–30 Hz) were computed.

### 2.3. Graph Theoretical Analysis

In this paper, the MI matrix was converted into an undirected binary graph by applying three network filtering schemes: absolute threshold *T*, mean degree *K*, and OMST method. Because there is no optimal way to select *T*, here the range of 0.15 < *T* < 0.45 (in step of 0.005) was selected for the four bands. It is well-known that the edge number in a graph has a great relationship with the values of *L* and *C*, and the edge number in the 2 graphs (SZ patients and healthy controls) will be different by applying a certain *T*. Therefore, in order to eliminate this effect, the *L* and *C* were calculated as a function of degree *K* (2 < *K* < 8, in step of 0.1).

Recently, Dimitriadis et al. [37, 38] proposed a novel data-driven topological filtering scheme based on OMST, which filters brain connectivity networks based on the optimization between the global efficiency of the network and the cost preserving its wiring. Here, we tried using the OMST method to filter the constructed brain networks and recomputed graph theoretical measures. After the MI matrix was converted into an undirected binary graph, the graph theoretical measures, such as characteristic path length *L* and cluster coefficient *C*, were computed. Detailed descriptions and calculation methods for *L* and *C* could be found in some previously published literature [39, 40].

The small-world network is characterized by a similar path length and higher cluster coefficient compared to a random network, that is, *γ* = *C*_real_/*C*_random_ > 1, *λ* = *L*_real_/*L*_random_ ≈ 1. And the small-world index could be defined as *σ* = *γ*/*λ*. For the small-world network, the *σ* is greater than 1. Here, in order to compute small-world indexes of experimental networks (SZ patients and healthy controls), after applying the OMST filtering scheme, 300 random networks were generated for each experimental network by using the Markov-chain algorithm [41, 42]. As a result, the mean small-world index *σ* of experimental networks (SZ patients and healthy controls) was computed.

### 2.4. Statistical Analysis

All statistical analyses were carried out using SPSS version 21.0 software (SPSS Inc., Chicago, IL). The Shapiro-Wilk test was used to test for normality of distribution. Task performance, such as execution time and error number, was statistically compared between SZ patients and healthy controls by using independent sample *t*-test. The differences of *C* and *L* between SZ patients and healthy controls for each value over a range of *T* or degree *K* were compared by using the Mann–Whitney *U* test. In addition, Pearson correlation analysis was applied to explore whether there existed correlation between *C* or *L* and task performance. *P* < 0.05 showed that a significant difference existed.

## 3. Results

The mean executive times of the TMT-B task for SZ patients and healthy controls were 123.85 ± 27.11 s and 75.93 ± 17.79 s, respectively. Independent sample *t*-test was used to analyze their statistical difference, and results indicated that the mean executive time of SZ patients was significantly longer than that of healthy controls (*P* = 0.000). The mean error numbers during TMT-B for SZ patients and healthy controls were 1.46 ± 1.98 and 0.31 ± 0.84, respectively, and statistical analysis implied that the mean error number of SZ patients was significantly larger than that of healthy controls (*P* = 0.001). We believed that the above behavioral results were mainly due to the cool executive dysfunction of SZ patients.

**Figure 1:**
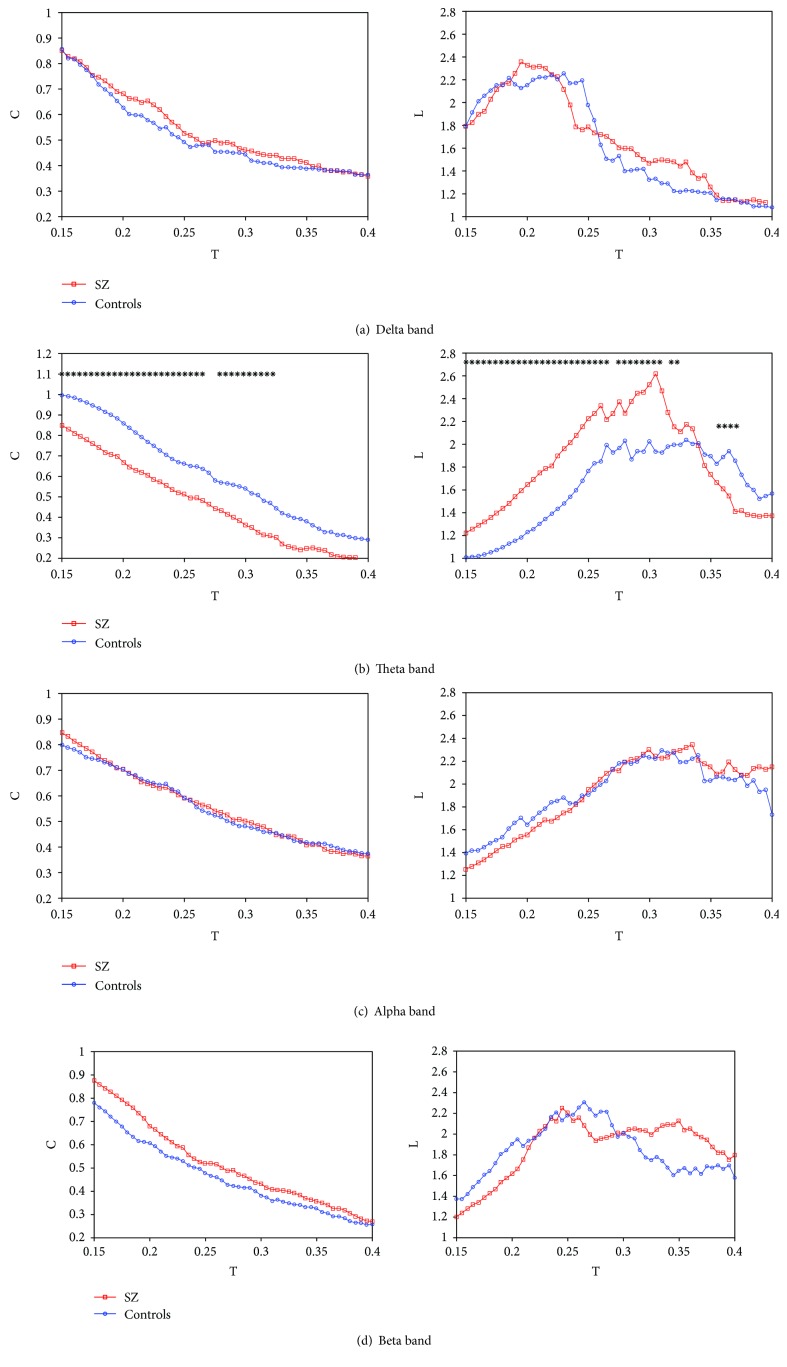
Mean cluster coefficient *C* and characteristic path length *L* for SZ patients (square) and healthy controls (circle) as a function of *T* in the delta band (a), theta band (b), alpha band (c), and beta band (d). The asterisks showed significant difference between SZ patients and healthy controls (*P* < 0.05).

Figure 1 showed the computed mean cluster coefficient *C* and characteristic path length *L* for SZ patients and healthy controls as a function of threshold *T* in the delta band (Figure 1(a)), theta band (Figure 1(b)), alpha band (Figure 1(c)), and beta band (Figure 1(d)). In all frequency bands, it was found that the mean cluster coefficient *C* decreased almost linearly with the increase of *T*. This was because more and more connectivity between the nodes in the graph was lost when threshold *T* increased. In addition, when *T* values were small, the characteristic path length *L* increased almost linearly with the increase of *T*, and this was because more and more connectivity between the nodes dropped out when threshold *T* increased, which increased the average path length between randomly selected nodes. When the threshold *T* reached a certain value, the characteristic path length *L* started to decrease. This phenomenon could be explained by the fact that the graph was divided into more than 2 subgraphs when *T* further increased and the resulted subgraphs were smaller than the original graph, which led to the decrease of mean *L*.

As shown in Figures 1(a), 1(c), and 1(d), there existed no significant difference between SZ patients and healthy controls for *C* and *L* in the delta, alpha, and beta bands in the range of 0.15 < *T* < 0.45 (in step of 0.005). As indicated in Figure 1(b), for a wide range of *T* (0.15 < *T* < 3.3), there always existed significant differences between SZ patients and healthy controls for *C* and *L* in the theta band. And the most significant difference for *C* and *L* occurred at *T* = 0.195 (Mann–Whitney *U* test, *U* = 40.000, *W* = 145.000, *P* = 0.008) and 0.305 (Mann–Whitney *U* test, *U* = 27.000, *W* = 132.000, *P* = 0.001), respectively.

**Figure 2:**
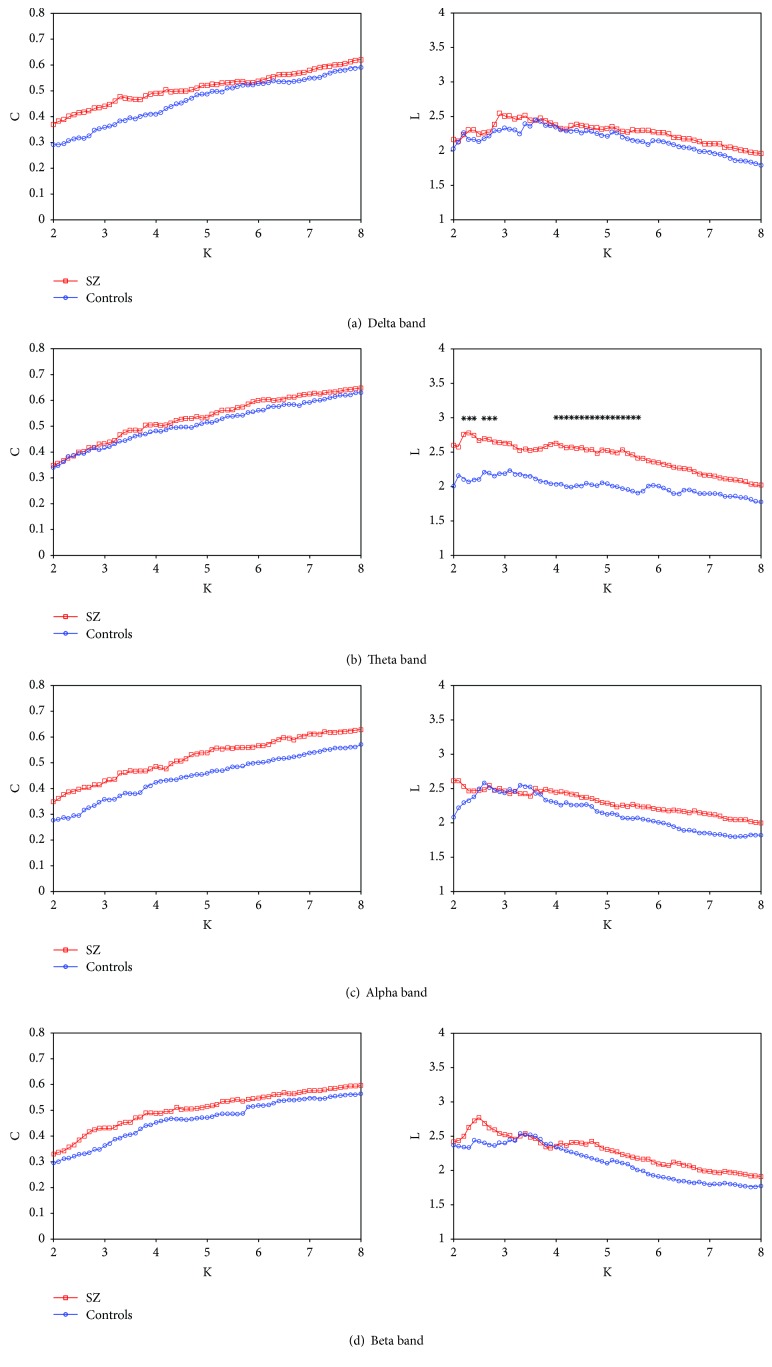
Mean cluster coefficient *C* and characteristic path length *L* for SZ patients (square), healthy controls (circle), ordered network (cross), and random network (triangle) as a function of *K* in the delta band (a), theta band (b), alpha band (c), and beta band (d). The asterisks showed significant difference between SZ patients and healthy controls (*P* < 0.05).

As shown in Figure 2, the mean cluster coefficient *C* and characteristic path length *L* for SZ patients and healthy controls were computed as a function of degree *K* in the delta band (Figure 2(a)), theta band (Figure 2(b)), alpha band (Figure 2(c)), and beta band (Figure 2(d)). For all frequency bands, it was found that the mean cluster coefficient *C* increased almost linearly with the increase of *K*. For the delta (Figure 2(a)), alpha (Figure 2(c)), and beta (Figure 2(d)) bands, there was no significant difference between SZ patients and healthy controls for *C* and *L*. As shown in Figure 2(b), there existed significant difference for *L* between SZ patients and healthy controls for wide ranges of 2.2 < *K* < 2.8 and 4.0 < *K* < 5.8 (*P* < 0.05), and the most significant difference occurred at *K* = 4.1 (Mann–Whitney *U* test, *U* = 37.000, *W* = 142.000, *P* = 0.005). However, there was no significant difference for *C* between SZ patients and healthy controls for the whole range of *K*.

**Figure 3:**
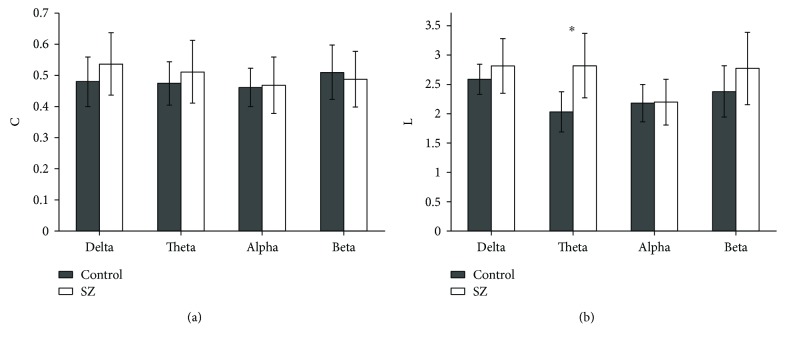
Mean cluster coefficient *C* (a) and characteristic path length *L* (b) in the four bands for SZ patients and healthy controls based on the OMST filtering scheme. The asterisk showed significant difference between SZ patients and healthy controls (*P* < 0.05).

The above two network thresholding schemes (absolute threshold and mean degree) are arbitrary thresholding schemes that might add bias for group and task comparisons and reduce the possibility of the reproducibility of the findings across studies from different research groups. So recently, Dimitriadis et al. [37, 38] proposed a novel data-driven topological filtering scheme based on OMST, which filters brain connectivity networks based on the optimization between the global efficiency of the network and the cost preserving its wiring. Here, the graph theoretical measures were recomputed by applying the OMST filtering scheme. As shown in Figure 3, the mean cluster coefficient *C* (Figure 3(a)) and characteristic path length *L* (Figure 3(b)) for SZ patients and healthy controls were computed based on the OMST scheme in the four bands. It is obvious that there was no significant difference for *C* between SZ patients and healthy controls in the four bands. However, significant difference existed only in the theta band for *L* between SZ patients and healthy controls (Mann–Whitney *U* test, *U* = 30.500, *W* = 135.500, *P* = 0.002), which was consistent with that of the mean degree *K* scheme (Figure 2(b)).

The small-world index could be defined as *σ* = *γ*/*λ*, where *γ* = *C*_real_/*C*_random_ and *λ* = *L*_real_/*L*_random_. After applying the OMST filtering scheme for experimental networks (SZ patients and healthy controls), 300 random networks were generated for each experimental network by using the Markov-chain algorithm [41, 42], and the corresponding *γ*, *λ*, and *σ* were computed. Results indicated that the small-world indexes of SZ patients and healthy controls were 3.148 ± 1.263 and 2.892 ± 1.475, respectively, and the small-world indexes of both groups were greater than 1, indicating that both groups had small-world network characteristics during the TMT-B task. However, it was found that there was no significant difference for the small-world index between the two groups (Mann–Whitney *U* test, *U* = 64.000, *W* = 169.000, *P* = 0.118).

**Figure 4:**
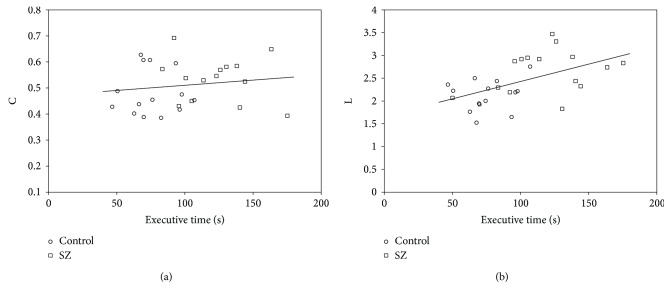
Pearson correlation analysis between *C* (a) or *L* (b) and task performance (executive time) in the theta band for SZ patients (square) and healthy controls (circle).

Pearson correlation analysis was applied to explore whether there existed correlation between *C* or *L* and the task performance (executive time). As indicated in Figure 4(a), the correlation coefficient between executive time and *C* was not significant for the combined SZ patients and healthy controls (*r* = 0.146, *P* = 0.448) or SZ patients alone (*r* = −0.186, *P* = 0.521).  Figure 4(b) showed that the *L* were positively correlated with executive times for the combined SZ patients and healthy controls (*r* = 0.507, *P* = 0.006), but not for SZ patients alone (*r* = 0.072, *P* = 0.412).

## 4. Discussion

Here, we studied the brain functional connectivity and its small-world topologic properties underlying cool executive dysfunction in first-episode SZ patients for the first time. We observed that changes of small-world network properties mainly appeared in the theta band, not in the delta, alpha, or beta band, and the SZ group was characterized by a longer characteristic path length *L* (having significant difference compared with the healthy group) and relatively higher cluster coefficient *C* (no significant difference compared with the healthy group) in the theta band, suggesting a less optimal organization of the brain network in SZ patients.

It was well-known that the characteristic path length *L* is defined as the average shortest paths for all possible pairs of nodes and stands for global efficiency of information integration across different brain areas. Our results showed that in the theta band, the *L* of SZ patients was significantly longer than that of healthy controls over a wide range of threshold *T* (Figure 1(b)), and this pattern was still present when *L* was calculated as a function of degree *K* (Figure 2(b)) or by using the OMST filtering scheme (Figure 3(b)), suggesting a more effective information integration and communication across different brain regions in healthy controls compared with SZ patients. In addition, some fMRI studies have reported fewer hubs (i.e., highly connected nodes) in SZ patients [43, 44], and such reduction in the number of highly connected nodes may explain the longer *L* for SZ patients in our study. Moreover, the cluster coefficient *C* was considered as a metric of the network segregation and of the local efficiency of information communication. It was indicated that the *C* of SZ patients was significantly smaller than that of healthy controls over the whole range of *T* (Figure 1(a)), suggesting that the local connections of networks in SZ patients were relatively spared. However, no significant difference for *C* occurred between SZ patients and healthy controls when *C* was calculated as a function of degree *K* (Figure 2(a)) or by applying the OMST filtering scheme (Figure 3(a)). It was well-known that the edge number in a graph has a great relationship with the values of *L* and *C*, and the edge number in the two graphs (SZ patients and healthy controls) will be different by applying a certain *T*. Therefore, it was necessary to compute *L* and *C* as a function of degree *K*, which ensured the same edge number in the two groups, and the resulted differences in *L* and *C* between the two groups would represent the differences of network configuration.

Although the above two network thresholding schemes (absolute threshold and mean degree) have been widely applied to threshold brain networks, they might add bias for group and task comparisons and reduce the possibility of the reproducibility of the findings across studies from different research groups. Therefore, in order to test the reproducibility of our results, we applied a novel data-driven topological filtering scheme based on the OMST proposed by Dimitriadis et al. [37, 38] to filter our constructed brain networks. Interestingly, we also found that significant difference existed only in the theta band for *L* between SZ patients and healthy controls (Figure 3(b)), which was consistent with that of the mean degree *K* scheme (Figure 2(b)). As described above, no matter which filtering scheme is used, our results always showed that SZ patients showed a significantly longer *L* compared to healthy controls. Therefore, the longer *L* in SZ patients cannot be due to the influence of other factors in the two groups and reflects a true disturbance in the brain network organization of this illness, which was consistent with some previously published literature [27–29].

We applied the OMST scheme to threshold experimental networks (SZ patients and healthy controls), and corresponding random networks were generated to compute the small-world index. For a small-world network, the *C* should be much larger than that of the random network and *L* should be close to that of a random network. Interestingly, the calculated small-world indexes of both groups were greater than 1, suggesting that both groups had small-world network characteristics in the theta band. However, the small-world index of SZ patients was larger than that of healthy controls, but no significant difference existed.

We also studied the correlations between *C* (Figure 4(a)) or *L* (Figure 4(b)) of the theta-band brain network and task performance (executive time). Previous literatures have reported that SZ patients need to spend more time to finish some executive tasks due to their executive dysfunction [3, 45], and our results indicated that the mean executive time of TMT-B task for SZ patients was significantly longer than that of healthy controls, which was consistent with these literatures.  Figure 4(b) showed that the *L* was positively correlated with executive times for the combined SZ patients and healthy controls, suggesting that the longer *L* would lead to the longer executive time of the TMT-B task. It was well-known that *L* reflects the global efficiency of information integration across different brain areas, and the *L* of SZ patients was significantly longer than that of healthy controls (Figure 3(b)). By taking all these considerations into account, we could infer that increased *L*, suggesting reduced global information integration ability, eventually led to increased executive time in SZ patients.

In recent years, a large number of studies have found that abnormal theta oscillation was closely related to SZ [46]. Increased theta activity was often observed in SZ patients during the rest state [47], whereas many studies showed reduced theta activity in SZ patients during various tasks [48]. In addition, the altered theta-band brain functional connectivity among different brain regions in SZ patients also has been confirmed in many literatures [10, 49]. The theta band was considered to play a key role in large-scale functional integration by combining the activities of various brain regions together [46, 50], and executive function that is involved in a range of higher-level cognitive processes relies on the integration of different brain areas for proper functioning [49]. The present study showed that there existed significant difference for small-world topologic properties between the two groups during the cool executive task only in the theta band, not in the other frequency bands, which supported the above-mentioned results. However, our study indicated that there was no significant difference for the small-world index in the theta band between first-episode SZ patients and healthy controls during the cool executive function task, which was in contrast with the study of Jhung et al. [26] in which the small-world index in the theta band of first-episode SZ patients during working memory task significantly decreased compared with that of healthy controls. This might suggest that the impairment degree of the cold executive function is much less than that of working memory function in first-episode SZ patients.

The present study has certain limitations. Firstly, in order to get more reliable conclusions, the sample size of subjects must be increased. Secondly, we studied the small-world topologic properties of SZ patients during only one cool executive task, that is, the TMT-B task, but it is not clear whether task difficulty influences the small-world topologic properties of SZ patients. Therefore, it is necessary and interesting to design cool executive tasks with different difficulty to study this issue in future work. Moreover, from a methodological point of view, our study converted functional connectivity based on MI into a binary graph, which would result in the loss of part of the information compared to the weighted graph.

## 5. Conclusions

Our results indicated that a less-optimal organization of the brain functional network in the theta band occurred in first-episode SZ patients compared with healthy controls. SZ patients owned a significantly longer characteristic path length *L* in the theta band no matter which filtering scheme is used, which suggested a disturbance in globally efficient communication between different brain areas in SZ patients. The present study combining functional connectivity and graph theory analysis provided helpful findings to reveal the pathophysiologic mechanisms underlying cool executive dysfunction in first-episode SZ patients.

## Data Availability

The data used to support the findings of this study are available from the corresponding author upon request.
